# Theragnosis for Duchenne Muscular Dystrophy

**DOI:** 10.3389/fphar.2021.648390

**Published:** 2021-06-03

**Authors:** Leonela Luce, Micaela Carcione, Chiara Mazzanti, Paula I. Buonfiglio, Viviana Dalamón, Lilia Mesa, Alberto Dubrovsky, José Corderí, Florencia Giliberto

**Affiliations:** ^1^ Laboratorio de Distrofinopatías, Cátedra de Genética, Facultad de Farmacia y Bioquímica, Universidad de Buenos Aires, Buenos Aires, Argentina; ^2^ Instituto de Inmunología, Genética y Metabolismo (INIGEM), CONICET—Universidad de Buenos Aires, Buenos Aires, Argentina; ^3^ Instituto de Investigaciones en Ingeniería Genética y Biología Molecular (INGEBI) “Dr. Héctor N. Torres”, CONICET, Buenos Aires, Argentina; ^4^ Instituto de Neurociencias, Fundación Favaloro, Buenos Aires, Argentina

**Keywords:** dystrophinopathies, duchenne muscular dystrophy (DMD), meta-analysis, theragnosis, mutagenic spectrum, nonsense, exon skipping, Latin America

## Abstract

Dystrophinopathies cover a spectrum of rare progressive X-linked muscle diseases, arising from *DMD* mutations. They are among the most common pediatric muscular dystrophies, being Duchenne muscular dystrophy (DMD) the most severe form. Despite the fact that there is still no cure for these serious diseases, unprecedented advances are being made for the development of therapies for DMD. Some of which are already conditionally approved: exon skipping and premature stop codon read-through. The present work aimed to characterize the mutational spectrum of *DMD* in an Argentinian cohort, to identify candidates for available pharmacogenetic treatments and finally, to conduct a comparative analysis of the Latin American (LA) frequencies of mutations amenable for available DMD therapies. We studied 400 patients with clinical diagnosis of dystrophinopathy, implementing a diagnostic molecular algorithm including: MLPA/PCR/Sanger/Exome and bioinformatics. We also performed a meta-analysis of LA’s metrics for DMD available therapies. The employed algorithm resulted effective for the achievement of differential diagnosis, reaching a detection rate of 97%. Because of this, corticosteroid treatment was correctly indicated and validated in 371 patients with genetic confirmation of dystrophinopathy. Also, 20 were eligible for exon skipping of exon 51, 21 for exon 53, 12 for exon 45 and another 70 for premature stop codon read-through therapy. We determined that 87.5% of DMD patients will restore the reading frame with the skipping of only one exon. Regarding nonsense variants, UGA turned out to be the most frequent premature stop codon observed (47%). According to the meta-analysis, only four LA countries (Argentina, Brazil, Colombia and Mexico) provide the complete molecular algorithm for dystrophinopathies. We observed different relations among the available targets for exon skipping in the analyzed populations, but a more even proportion of nonsense variants (∼40%). In conclusion, this manuscript describes the theragnosis carried out in Argentinian dystrophinopathy patients. The implemented molecular algorithm proved to be efficient for the achievement of differential diagnosis, which plays a crucial role in patient management, determination of the standard of care and genetic counseling. Finally, this work contributes with the international efforts to characterize the frequencies and variants in LA, pillars of drug development and theragnosis.

## Introduction

Muscular Dystrophies (MDs) are hereditary disorders that cause weakness and progressive degeneration of skeletal muscles. These diseases are caused by molecular alterations in a wide range of genes that encode proteins that participate in the stability, maintenance, repair, regeneration and proper functioning of muscle fibers (Wallace and McNally, 2009). Although DMD clinical features are quite typical for the trained physician, there are other less frequent forms of MDs with similar clinical characteristics such as sarcoglycanopathies, laminopathies and other forms of LGMD. Therefore, the clinical diagnosis can be misled by these overlapping features, turning the molecular diagnosis into a crucial tool for the achievement of a differential diagnosis.

Dystrophinopathies are the most frequent form of MDs among the pediatric population. These are X-linked recessive diseases caused by pathogenic variants in the *DMD* gene (OMIM ID: 300377) (Hoffman et al., 1987; Koenig et al., 1988). Although in theory dystrophinopathies can be subdivided into three distinctive clinical conditions, Duchenne muscular dystrophy (DMD), Becker muscular dystrophy (BMD), and DMD-associated dilated cardiomyopathy (DCM), they actually entail a continuous spectrum of muscle diseases (Darras et al., 2000; Brandsema and Darras, 2015). DMD is the most prevalent and severe pediatric form of MD, with an incidence of 1:3500–5000 male births (Mendell et al., 2012). It is characterized by progressive muscle-waste, which leads to disability and premature death (Aartsma-Rus et al., 2017). On the other hand, BMD affects 1:18.000 born males and has a milder symptomatology pattern and/or slower progression rate than DMD.

The genotype/phenotype correlation relies on the impact of the molecular alteration on dystrophin function. DMD is mainly associated with mutations leading to complete absence of functional dystrophin, such as frameshift or nonsense variants. Instead, BMD is caused by a decrease in the amount or function of dystrophin, as it would be the case of in-frame variants (Guiraud et al., 2015). Nonetheless, in some cases, the phenotype predicted on the basis of molecular alterations detected at genomic level do not correlate with the observed clinical picture. This would be the case of patients carrying out-of-frame or nonsense variants but showing a mild progression of the disease, which could be explained by an endogenous exon skipping restoring the reading frame or avoiding the premature stop codon, respectively.

Mutational spectrum of the *DMD* gene comprise mainly copy number variants (CNVs), such as deletions (∼68%) or duplications (∼11%) of one or more exons, and small molecular alterations in the remaining ∼20% (Aartsma-Rus et al., 2016). In addition, around half of small sequence variants are nonsense substitutions.

Accurate molecular diagnosis, given by the identification and precise characterization of deleterious variants, is crucial for dystrophinopathy patients to confirm the clinical presumptive diagnosis, to access to the specific and optimal standard of care (Bushby et al., 2010) and determine eligibility for the available pharmacogenetic treatments. For example, molecular confirmation of dystrophinopathy determines applicability of corticosteroid therapy, as DMD is one of the MDs showing fruitful results from this treatment (Albuquerque et al., 2014; Bello et al., 2015). On the other hand, molecular diagnosis plays a key role in family planning and, therefore, prevention.

Despite the fact that there is still no cure for these serious diseases, unprecedented advances are being made for the development of therapies for DMD. Hitherto, three mutation specific treatments already have conditional approval: premature stop codon read-through (Ataluren) by the European Medicines Agency (EMA) and exon skipping for exon 51 (Eteplirsen) and exon 53 (Golodirsen) by the Food and Drug Administration (FDA) (Haas et al., 2015; Mah, 2018).

The rationale of “exon skipping” is to restore the *DMD* reading frame by the removal of one or several exons adjacent to any of the deletion’s borders, which is accomplished by targeting regulatory splice sites in the pre-mRNA (Syed, 2016; Kinane et al., 2018). Therefore, the resulting spliced transcript might generate a partially functional dystrophin, albeit internally deleted and quantitatively reduced, capable of shifting the patient’s severe phenotype into a milder one (Syed, 2016; Kinane et al., 2018). Apart from the previously mentioned exon skipping for exon 51 and exon 53, which apply to 10–15% and 8–10% of DMD patients respectively, antisense oligonucleotides to target exon 45 (Casimersen) are now pursuing FDA’s approval.

On the other hand, the principle behind Ataluren is the endogenous process known as “stop codon suppression or readthrough”, which entails the recognition of stop codons by a near-cognate aminoacyl-tRNA (Keeling and Bedwell, 2011). The efficacy of the suppression process depends on several conditions: the innate readthrough capacity of each stop codon (UGA > UAG > UAA), the sequence surrounding the termination codon and the functionality of the incorporated amino acid (Miller and Pearce, 2014). This therapy specifically applies to patients carrying *DMD* nonsense mutations (10–15%).

In addition, under the name of dystrophin restoration therapies are included the gene-transfer strategy, which incorporates short versions of the *DMD* gene but encoding functional mini/micro-dystrophins, and the *DMD* gene-editing approach, that applies CRISPR-Cas9 technology to correct the molecular alteration carried by each individual (Duchêne et al., 2018; Verhaart and Aartsma-Rus, 2019; Lim et al., 2020; Mendell et al., 2020). Furthermore, gene-transfer therapies for other types of muscular dystrophies (*CAPN3*, *SGCB*, *SGCA, DYSF*, *SGCG* and *ANO5*) are burgeoning (Gene Therapy Engine; Chu and Moran, 2018)^1^.

On the other hand, one of the pillars for drug development and theragnosis is the information regarding the frequency and types of molecular alterations that take place in a certain gene. However, this knowledge principally comes from Europe and the United States, as little is known about the Latin American frequencies, which is also true for *DMD*.

Therefore, the present work has three major aims. Firstly, the characterization of the mutational spectrum of the *DMD* gene in an Argentinian dystrophinopathy cohort. Secondly, the identification of candidate patients for the available pharmacogenetic treatments for DMD. Finally, the conduction of a comparative analysis of the Latin American frequencies of the mutations amenable for the available DMD therapies.

## Materials and Methods

### Patients and Samples

A cohort of 400 boys with presumptive clinical diagnosis of dystrophinopathy was referred to our laboratory in pursuit of differential molecular diagnosis. The criteria followed for the clinical diagnosis was the one described in Birnkrant et al., 2018. The algorithm began with the clinical assessment. Clinical suspicion of DMD arose in cases with DMD family history or based on the observation of progressive muscular weakness, Gowers sign, calf muscle pseudohypertrophy, difficulty at climbing stairs, waddling gait and/or toe walking. The second step was the determination of the CK level, followed by molecular studies. If no pathogenic variant is found by genetic testing the guideline recommends a muscle biopsy.

Whole blood was drawn by venipuncture with 5% ethylene-diamine tetraacetic acid (EDTA) as anticoagulant for all study subjects. Genomic DNA was isolated using the cetyl-trimethyl-ammonium bromide (CTAB) method (Murray and Thompson, 1980). DNA concentration and quality were measured by absorbance at 260 nm and by the ratio of A260 nm/A280 nm, respectively. All samples were stored at −20°C.

The protocol was approved by the Institutional Review Board. Informed consent was obtained for all study subjects prior to the molecular studies.

### Multiplex Ligation-dependent Probe Amplification (MLPA)

The commercially available MLPA kit for the *DMD* gene (Salsas PO34–PO35) was used to screen for gene deletions/duplications (Schwartz and Dunø, 2004; Gatta et al., 2005; Janssen et al., 2005). Reactions were carried out according to the manufacturer’s recommendations [MRC-Holland, Amsterdam, Netherlands (www.mlpa.com)]. Products were analyzed using a fragment analyzer sequencer (ABI 3730XL; Applied Biosystems, Foster City, California) and 500Liz as internal size standard. Data analysis was performed using Coffalyser (MRC-Holland, Amsterdam, Netherlands) and GeneMarker V2.2.0 (Softgenetics, State College, Pennsylvania) software. Wild-type, deleted, and duplicated controls were included in all reactions. Following the best practice guidelines for genetic testing for dystrophinopathies, cases with single-exon deletion were confirmed by PCR and/or Sanger sequencing (Fratter et al., 2020).

### Whole Exome Sequencing (WES)

WES was carried out by Macrogen Services (Republic of Korea). Exome libraries were constructed by hybridization capture with the Agilent SureSelect V4/V5/V6 Target Enrichment Kits (Agilent Technologies, Santa Clara, United States). WES was performed on the Illumina HiSeq4000/NovaSeq6000 platforms (Illumina, San Diego, United States), following the manufacturer’s recommendations. FASTQ sequencing files were aligned to the Human Reference Genome hg19 from UCSC (original GRCh37 from NCBI, Feb. 2009) applying Burrows-Wheeler Alignment Tool (BWA−0.7.12). Analysis proceeded using Picard (picard-tools-1.130) and Genome Analysis Toolkit (GATK3.v4). Finally, variant annotation was carried out applying SnpEff (SnpEff_v4.1g), dbSNP database (version 142), 1000Genomes phase 3, ClinVar database (version 05/2015) and ESP database (ESP6500SI_V2). Furthermore, in order to determine the coverage, coverage depth and the quality of the reads, bam files were analyzed using the Integrative Genomics Viewer (IGV) software (Broad Institute, University of California, United States).

### Selection of Disease-Associated Candidate Variants

The screening of disease-associated variants from the NGS results started with the analysis of the *DMD* gene. When no *DMD* disease associated variants were identified, we broadened the analysis to genes associated with other monogenic neuromuscular disorders (NMDs), beginning with muscular dystrophies, group 1 of “The Gene table of Neuromuscular disorders” (Benarroch et al., 2019). When no disease-associated variants were found, we extended the search to all the groups listed in the table previously mentioned (Benarroch et al., 2019).

The detected sequence variants were classified according to the standards and guidelines of the American College of Medical Genetics and Genomics (ACMG) and the Association for Molecular Pathology (Richards et al., 2015). Nomenclature of the identified variants was achieved following the HGVS standards (Dunnen et al., 2016). The classification of variants was performed on the basis of the information gathered from: 1) Type and effect of the molecular alteration; 2) Population data from 1000Genomes and gnomAD (https://gnomad.broadinstitute.org/); 3) disease/gene specific databases, such as Leiden open variation database (LOVD) (http://www.lovd.nl/3.0/home/) and ClinVar (https://www.ncbi.nlm.nih.gov/clinvar/); 4) *In silico* predictive analysis: PolyPhen-2 (http://genetics.bwh.harvard.edu/pph2/), SIFT (http://sift.jcvi.org/), Mutation Taster (http://www.mutationtaster.org/), Mutation Assessor (http://mutationassessor.org/r3/), CADD (http://cadd.gs.washington.edu/), UMD Predictor (http://umd-predictor.eu/analysis.php), Human Splicing Finder (https://www.genomnis.com/access-hsf), etc.; 5) Phenotypic features; 6) Familial segregation; and, 7) Bibliographic reports of functional assays.

### Polimerase Chain Reaction (PCR)-Sanger Sequencing

Every disease associated or likely pathogenic variant identified by WES and single exon deletion observed by MLPA was corroborated by PCR-Sanger sequencing. Also, this technique was employed for the analysis of patients with known familial causative small molecular alteration. Primer sequences and PCR conditions were obtained from the Leiden muscular dystrophy site [Leiden muscular dystrophy webpages (www.dmd.nl)]. All PCR reactions were performed in a thermal cycler (Veriti; Applied Biosystems, Foster City, California). PCR amplicons were analyzed by 2% agarose (Genbiotech SRL) gel electrophoresis in 1X TBE buffer and dyed with GelRed™ (Biotium). Positive controls (wild-type DNA) and negative controls (no DNA) were included in all reactions. The exons were sequenced using both PCR primers and the reaction products were analyzed using a DNA analyzer (ABI 3730 XL; Applied Biosystems, Foster City, California). The quality of the obtained sequence was determined using FinchTV software (Geospiza, Seattle, United States) and the results were analyzed by comparison with the GenBank sequence of the *DMD* muscular isoform (Dp427m, NM_004006.3).

### Analysis of Exonic Targets for Exon Skipping

In order to establish the most frequent targets for exon skipping in our cohort, we selected a subset of 112 patients carrying out-of-frame deletions in the *DMD* gene. Duplications were excluded from this analysis as MLPA results do not provide information about the location of the duplicated exons nor the direction in which they were inserted. According to the GenBank sequence of the dystrophin muscular isoform (Dp427m, NM_004006.3), we determined for each deletion the minimum number of exons, both at the 5′ and 3’ borders of the molecular alteration, that could be skipped in order to restore the reading frame. This analysis was not restricted to the exons targeted by the available or underdevelopment therapies.

### Meta-Analysis of Latin America’s Metrics for Duchenne Muscular Dystrophy Available Therapies

In order to determine the frequency of candidate patients for the available therapies for each Latin American country, we performed a systematic review of the literature regarding molecular diagnosis of dystrophinopathies.

**FIGURE 1:**
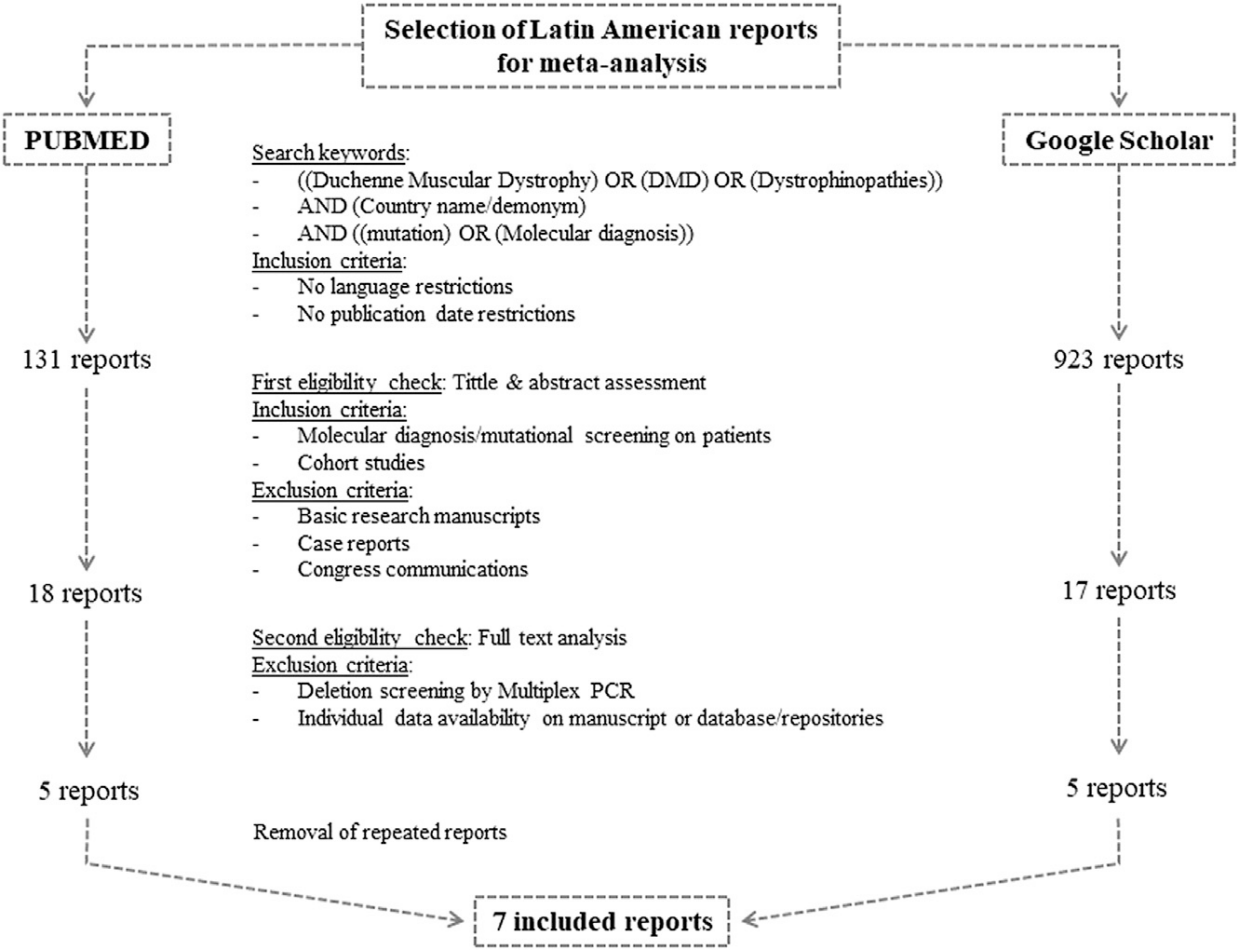
Flow chart of search and selection algorithm of Latin American reports for the meta-analysis.

The study was carried out following the “Preferred Reporting Items for Systematic reviews and Meta-Analyses” (PRISMA) guidelines (Page et al., 2021). Figure 1 summarizes the search and selection process. The search was conducted in PubMed from the National Library of Medicine (National Center for Biotechnology Information—NCBI) and in Google Scholar from Google (White, 2020). The following keywords were used to browse in both search engines [(Duchenne muscular dystrophy) OR (DMD) OR (Dystrophinopathies)] AND (Country name/demonym) AND [(mutation) OR (molecular diagnosis)]. We applied no publication date nor language restrictions. The last search was performed on November 30, 2020.

Three authors (LL, MC, and CM) independently carried out the study selection from the retrieved manuscripts. Firstly, potentially eligible reports were identified by assessing their title and abstract. At this stage, we included cohort studies and thesis conducting molecular diagnosis or mutational screening on patients with clinical presumptive diagnosis of dystrophinopathies. Manuscripts regarding basic research, case reports and congress communications were excluded.

The second eligibility step included the review of the full text. We excluded reports implementing multiplex-PCR, given that the exact deletion borders might not have been determined and that it resembles an underestimation of the amount of deletions. Moreover, so as to calculate the frequencies using a common criteria, we only considered manuscripts including the results of each individual or that had their results submitted on public repositories or databases such as the Leiden open variation database (LOVD) (https://www.lovd.nl/).

Once we had the selected reports, we manually extracted the following information: the employed molecular techniques, total amount of analyzed patients, the amount of individuals with genetic confirmation of dystrophinopathy, unrelated patients carrying deletions, unrelated individuals with deletions amenable by exon skipping of exon 45, 51 or 53, unrelated boys carrying small variants in *DMD* and unrelated patients with nonsense variants.

Additionally, so as to compare the calculated Latin American frequencies with the well-known and highly regarded frequencies from Europe and the United States, we conducted the screening described above with minor modifications for Spain, Italy, Portugal and the United States. We selected the above mentioned European countries on the basis of the most relevant migratory waves of the Latin American history. Granted that these four countries have been providing state of the art molecular diagnosis for dystrophinopathies for many years, the amount of available reports considerably exceeded the Latin American ones. Therefore, we decided to restrict the publication date (2005–2020) and select only one report per country, opting for the latest and/or the one with the largest cohort with available individual data.

## Results

### Molecular Diagnosis and Selection of Candidate Patients

**FIGURE 2:**
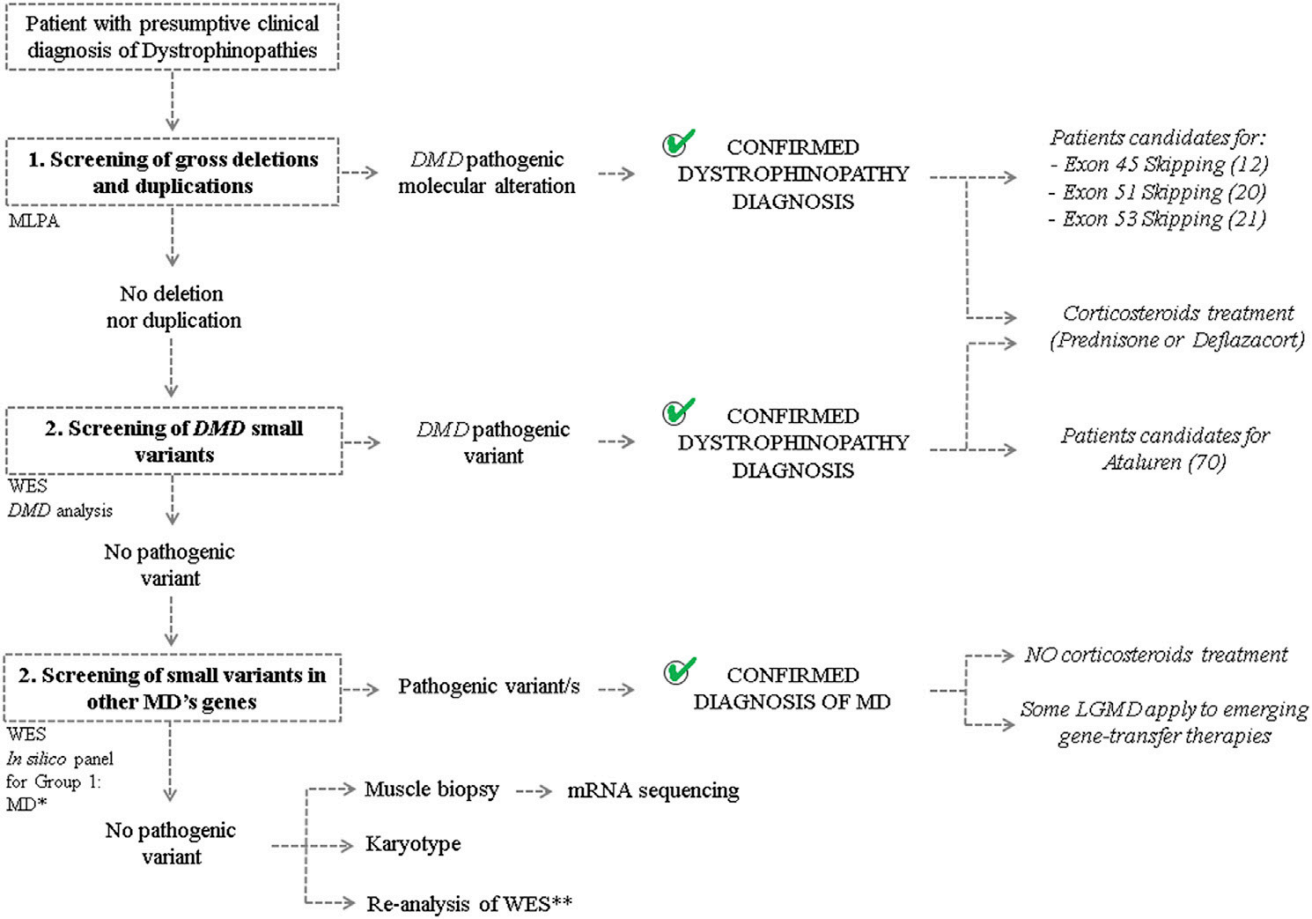
Molecular algorithm The figure shows the workflow carried out to achieve differential diagnosis of patients with clinical suspicion of Dystrophinopathy and to determine candidates for mutation-specific therapies. “MLPA”: multiplex ligation-dependent probe amplification. “WES”: whole exon sequencing. “MD”: Muscular Dystrophy. ^*^ *In silico* panel was created considering genes associated with the development of MDs (Group 1) according to the “Gene table of neuromuscular disorders (nuclear genome)” and its annual updates. ^**^Re-analysis of the WES results on the basis of the discovery of new genes involved with MDs and the revision of the ACMG classification of sequence variants.

From the studied cohort, dystrophinopathy clinical diagnosis could be confirmed in 371 from 400 analyzed patients. The employed molecular algorithm, based on the best practice guidelines for genetic testing for dystrophinopathies and the characteristics of each case (familial/sporadic case, known/unknown causative mutation and type of molecular alteration), reached a detection rate of 92.8% (Figure 2). Granted that we already had the WES results of the 29 patients without identified mutation in *DMD,* we broadened the screening of pathogenic variants to genes associated with other muscular dystrophies (Group 1). This extended algorithm allowed us to provide a differential diagnosis to other 17 patients and, also, to increase the detection rate to 97%. These patients showed overlapping symptoms with DMD/BMD but turned out to be principally affected by limb-girdle muscular dystrophies, as we found disease causing variants in the following genes: *FKRP* (4)*, SGCA* (2)*, SGCG* (2)*, SGCB* (1)*, CAPN3* (1)*, FKTN* (1)*, POMT2* (2)*, SYNE1* (1)*, COL6A1* (1)*, COL6A3* (1) and *PHKA1* (1). In the remaining 12 patients, we proceeded with the screening of all the other groups of NMD. However, we could not identify any disease associated variants.

Given the broadening of the analysis of WES results, we have identified two patients from our cohort carrying pathogenic or likely pathogenic molecular alterations in two MDs causing genes. Patient one presented an intronic variant in *DMD* (NM_004006.3:c.1332-9A > G), mainly reported as pathogenic in LOVD and ClinVar and probed to affect the splicing process. However, he also carried an heterozygous missense variant in *CAPN3* (NM_000070.2:c.1303G > A; NP_000061.1:p(Glu435Lys), classified as pathogenic in LOVD and as likely pathogenic/pathogenic in ClinVar. Patient two showed two variants in *DMD* and a heterozygous variant in *SYNE1*. In *DMD* gene, not only did he carry a splice site variant (NM_004006.3:c.9975-2A > T) but also a missense molecular alteration (NM_004006.3:c.10010G > A; NP_003997.2:p.Cys3337Tyr), both reported in LOVD database in a single occasion and classified as pathogenic and likely pathogenic, respectively. As for *SYNE1*, he presented a novel frameshift deletion [NM_182961.3:c.7310del; NP_892006.3:p(Gly2437Valfs*6)].

On the other hand, as it was mentioned above, the identification of the disease causative molecular alteration contributes to the selection of the suitable treatment for each individual (Figure 2). Firstly, the 371 patients with genetic confirmation of dystrophinopathy resulted candidates for corticosteroid treatment to ameliorate the inflammation and improve muscle strength and function. Moreover, regarding the available mutation-specific therapies for Dystrophinopathy, the precise characterization of the *DMD* mutation allowed us to determine that 20 patients were candidates for exon skipping of exon 51, 21 for exon 53 and 12 for exon 45, while another 70 were eligible for premature stop codon read-through therapy (Table 1). Alternatively, molecular diagnosis prevented the unnecessary and ineffective corticosteroid treatment of 17 patients diagnosed with other MDs. However, this differential diagnosis enabled us to determine that six patients were candidates for gene-transfer therapies for LGMD (2 LGMD2D—*SGCA*, 2 LGMD2C—*SGCG*, 1 LGMD2E—*SGCB* and 1 LGMD2A—*CAPN3*).

**TABLE 1:** Nonsense variants characterization.

Lab identification	Patients*	Nonsense variant (HGVS, c./p.)	Exon	Dys domains	WT aa	WT codón	Stop codon	DNA subst mut
#392	1	c.433C > T/p.(Arg145*)	6	Actin binding	Arg	CGA	**T**GA	Transition
#56	1	c.620T > G/p.(Leu207*)	7	Actin binding	Leu	TTA	T**G**A	Transversion
#598	1	c.701C > G/p.(Ser234*)	8	Actin binding	Ser	TCG	T**G**A	Transversion
#620	1	c.826C > T/p.(Gln276*)	8	Actin binding	Gln	CAA	**T**AA	Transition
#104	1	c.907C > T/p.(Gln303*)	9	Central rod	Gln	CAG	**T**AG	Transition
#586	1	c.1132C > T/p.(Gln378*)	10	Central rod	Gln	CAG	**T**AG	Transition
#246	1	c.1388G > A/p.(Trp463*)	12	Central rod	Trp	TGG	T**A**G	Transition
#362	1	c.1793C > G/p.(Ser598*)	15	Central rod	Ser	TCA	**T**GA	Transversion
#461	1	c.1777C > T/p.(Gln593*)	15	Central rod	Gln	CAA	**T**AA	Transition
#307	1	c.1928G > A/p.(Trp643*)	16	Central rod	Trp	TGG	T**A**G	Transition
#619	1	c.2032C > T/p.(Gln678*)	17	Central rod	Gln	CAG	**T**AG	Transition
#288	1	c.2270C > G/p.(Ser757*)	18	Central rod	Ser	TCA	T**G**A	Transversion
#132	1	c.2317A > T/p.(Lys773*)	19	Central rod	Lys	AAG	**T**AG	Transversion
#110/#725/#824	3	c.2407C > T/p.(Gln803*)	20	Central rod	Gln	CAA	**T**AA	Transition
#326	1	c.3151C > T/p.(Arg1051*)	20	Central rod	Arg	CGA	**T**GA	Transition
#303	1	c.2440G > T/p.(Glu814*)	20	Central rod	Glu	GAA	**T**AA	Transversion
#775	1	c.2566C > T/p.(Gln856*)	20	Central rod	Gln	CAA	**T**AA	Transition
#773	1	c.2414C > G/p.(Ser805*)	20	Central rod	Ser	TCA	T**G**A	Transversion
#723	1	c.2626G > T/p.(Glu876*)	21	Central rod	Glu	GAA	**T**AA	Transversion
#762/#695	2	c.2991C > G/p.(Tyr997*)	23	Central rod	Tyr	TAC	**T**AG	Transversion
#394/#460	2	c.3151C > T/p.(Arg1051*)	23	Central rod	Arg	CGA	**T**GA	Transition
#717	1	c.3136C > T/p.(Gln1046*)	23	Central rod	Gln	CAA	**T**AA	Transition
#125	1	c.3742C > T/p.(Gln1248*)	27	Central rod	Gln	CAG	**T**AG	Transition
#686	1	c.4108C > T/p.(Gln1370*)	30	Central rod	Gln	CAG	**T**AG	Transition
#258	1	c.4375C > T/p.(Arg1459*)	32	Central rod	Arg	CGA	**T**GA	Transition
DMD191	1	c.4499C > A/p.(Ser1500*)	32	Central rod	Ser	TCA	T**A**A	Transversion
#675/#677	2	c.4729C > T/p.(Arg1577*)	34	Central rod	Arg	CGA	**T**GA	Transition
#603	1	c.4820T > A/p.(Leu1607*)	34	Central rod	Leu	TTG	T**A**G	Transversion
#639	1	c.5530C > T/p.(Arg1844*)	39	Central rod	Arg	CGA	**T**GA	Transition
#649	1	c.6254G > A/p.(Trp2085*)	43	Central rod	Trp	TGG	T**A**G	Transition
#710	1	c.6715G > T/p.(Glu2239*)	46	Central rod	Glu	GAA	**T**AA	Transversion
#303/#338	2	c.6973C > T/p.(Gln2325*)	48	Central rod	Gln	CAG	**T**AG	Transition
#769	1	c.7010T > G/p.(Leu2337*)	48	Central rod	Leu	TTA	T**G**A	Transversion
#774	1	c.7657C > T/p.(Arg2553*)	52	Central rod	Arg	CGA	**T**GA	Transition
#465	1	c.7792C > T/p.(Gln2598*)	53	Central rod	Gln	CAG	**T**AG	Transition
#508	1	c.7750C > T/p.(Gln2584*)	53	Central rod	Gln	CAA	**T**AA	Transition
#689	1	c.8098A > T/p.(Lys2700*)	55	Central rod	Lys	AAG	**T**AG	Transversion
#285	1	c.8608C > T/p.(Arg2870*)	58	Central rod	Arg	CGA	**T**GA	Transition
#623	1	c.8774G > A/p.(Trp2925*)	59	Central rod	Trp	TGG	T**A**G	Transition
#483	1	c.8944C > T/p.(Arg2982*)	60	Central rod	Arg	CGA	**T**GA	Transition
#295	1	c.9337C > T/p.(Arg3113*)	64	Cysteine-rich	Arg	CGA	**T**GA	Transition
#194	1	c.9459T > A/p.(Cys3153*)	65	Cysteine-rich	Cys	TGT	TG**A**	Transversion
#673	1	c.9474T > G/p.(Tyr3158*)	65	Cysteine-rich	Tyr	TAT	TA**G**	Transversion
#542/#495/#700	3	c.9568C > T/p.(Arg3190*)	66	Cysteine-rich	Arg	CGA	**T**GA	Transition
#617	1	c.9802C > T/p.(Gln3268*)	67	Cysteine-rich	Gln	CAA	**T**AA	Transition
#196	1	c.9928C > T/p.(Gln3310*)	68	Cysteine-rich	Gln	CAG	**T**AG	Transition
#469/#437/#753/#846	4	c.10108C > T/p.(Arg3370*)	70	Cysteine-rich	Arg	CGA	**T**GA	Transition
#250	1	c.10141C > T/p.(Arg3381*)	70	Carboxy-terminal	Arg	CGA	**T**GA	Transition
#854	1	c.10171C > T/(p.Arg3391*)	70	Carboxy-terminal	Arg	CGA	**T**GA	Transition

Lab, laboratory; Patients, number of non-related patients with the same nonsense; Nonsense variant (HGVS, c./p.), HGVS-nomenclature (https://varnomen.hgvs.org/); p, (protein); c, (coding DNA) (Dp427m, NM_004006.3); WT aa, Wild type amino acid; WT Codón, Wild type codon, in bold the base implicated in the substitution; Dys Domains, dystrophin domains; DNA subst mut, DNA substitution mutations.

### Argentinian Duchenne Muscular Dystrophy Mutagenic Spectrum and Analysis of Exonic Targets for Exon Skipping

**FIGURE 3:**
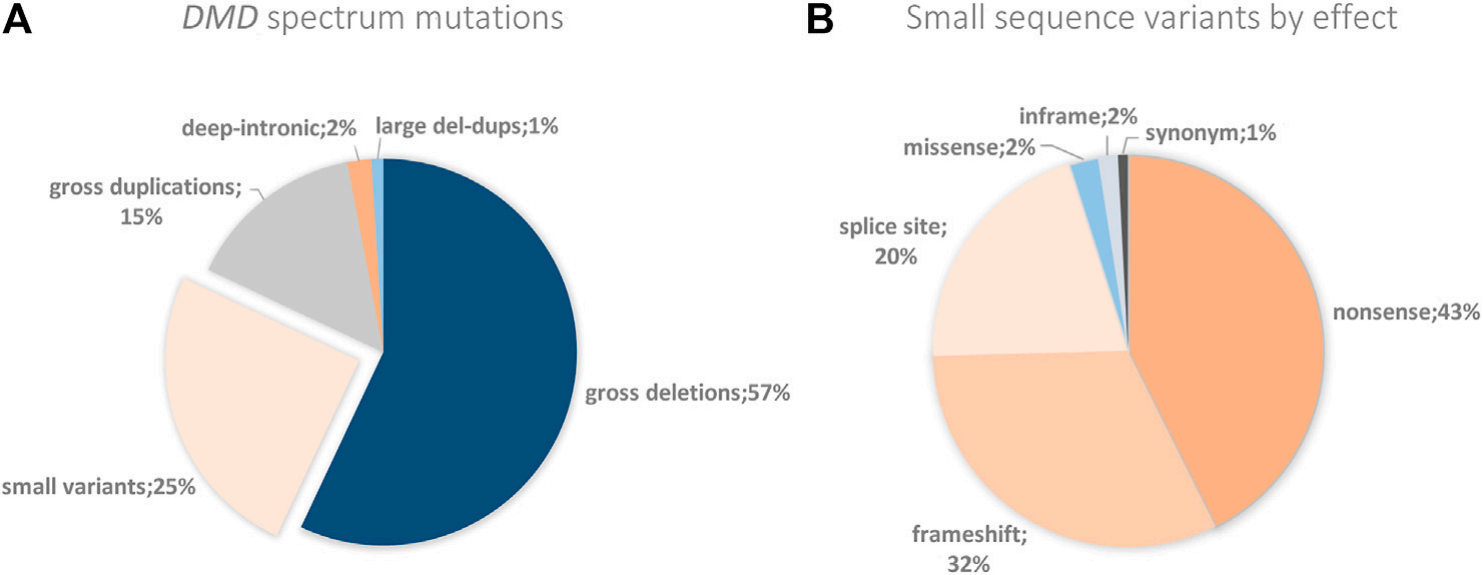
DMD spectrum mutations and small variants by effect. **(A)** The figure shows the *DMD* percentages of the different genetic alterations found in the Argentine cohort. **(B)** *DMD* percentages of the small variants by their effect found in the Argentine cohort.

So as to collaborate with the international efforts that aim to determine mutation frequencies from Latin America, we used our results to establish the *DMD* mutagenic spectrum for the Argentinian affected population. As expected, CNVs were the most frequent type of molecular alterations taking place in *DMD*, accounting for 71.5% of cases. Deletions of one or more exons were the major contributors of CNVs, being detected in 56.6% of cases, while duplications of one or more exons were found in 14.9%. CNVs were followed by small pathogenic sequence variants, which were identified in 25.4% of cases (Figure 3A). According to the classification by effect of the sequence variants, the three types most commonly found were nonsense (42.6%), followed by frameshift (32%) and splice site variants (20.5%) (Figure 3B). Furthermore, not only have we detected a small fraction of patients carrying a deletion and a duplication in the same allele, but also some non-contiguous duplications.

**FIGURE 4:**
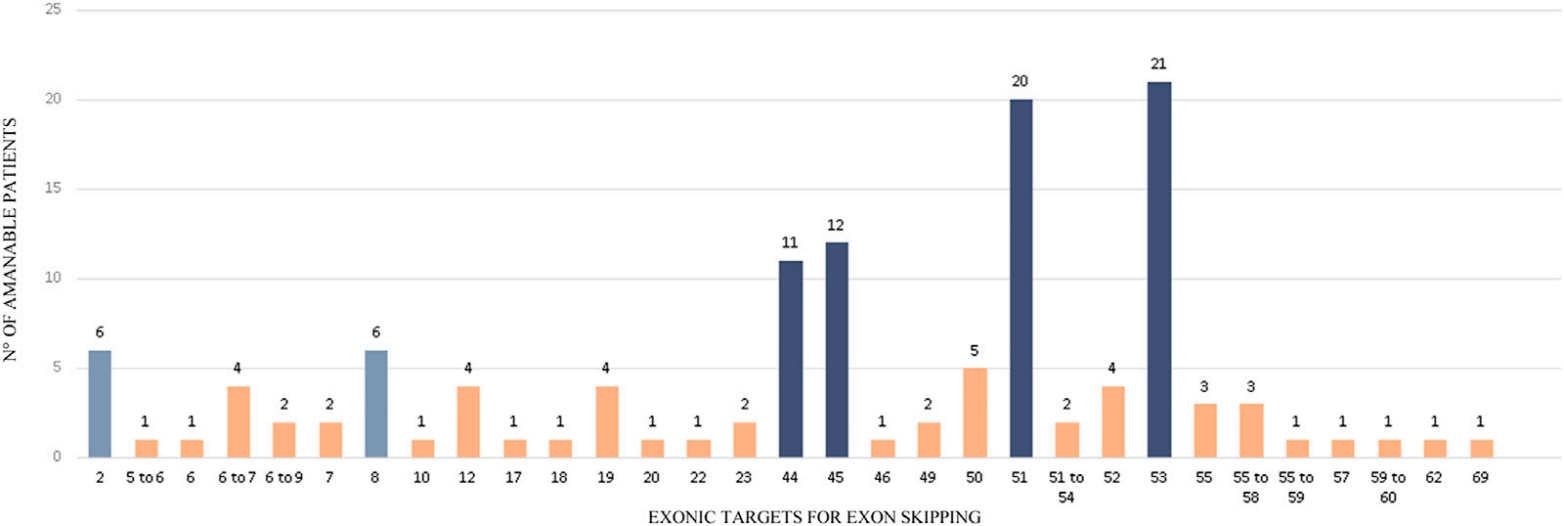
Exonic targets for Exon skipping. The figure shows the targets for exon skipping that could restore the reading frame of a subset of 112 patients carrying out-of-frame deletions in *DMD*. 14 mutations can be corrected by two different Exon Skipping strategies.

On the other hand, we wondered which were the most useful exonic targets for exon skipping in our cohort. To answer this query, we only took into account the 112 out-of-frame deletions identified. The putative single or multiple exonic targets that would restore the reading frame of the 112 deletions are depicted in Figure 4. The skipping of only one exon could restore the reading frame of 87.5% of patients, whereas the remaining 12.5% would require multiple-exon skipping. The deletions of 14 patients can even be corrected by two different single-exon skipping strategies, as removing the 5′ or 3’ exons adjacent to the alteration can turn it into an in-frame mutation.

As it was expected, most of the identified molecular alterations (47.3%) would restore the reading frame by skipping exons 45, 51 or 53, that is to say that they are eligible for the already available therapies. They are followed by exon 44, which applies to 9.8% of the out-of-frame deletions. It is worth mentioning that exon skipping of exon 44 is currently under preclinical test. Exon skipping strategies targeting 50 and 52, which are also undergoing preclinical trials, only probed to be eligible for 4.5 and 3.4% of the identified mutations, respectively. Strikingly, we found that 5.4% of the mutations from our cohort are amenable for exon skipping of exon 2 or exon 8, as all these patients carried the same deletion of exons 3 to 7 (Figure 4).

### Characterization of Nonsense Variants

Ataluren is the drug developed to enable ribosomal read-through of premature stop codons in nonsense mutations for Duchenne patients. From the total cohort analyzed we identified 70 patients with nonsense variants, who were candidates for Ataluren drug. We wonder if the subtypes of nonsense variants could affect Ataluren effectiveness, therefore we proposed to characterize the diversity of nonsense in an Argentine cohort. We described the different types of nonsense found, the number of times the same variant was observed in unrelated patients, and the exons and protein domains affected. Furthermore, we analyzed which was the wild-type amino acid that switched to a premature stop codon, and finally, the codon position of the transition/transversion (Table 1).

From the 70 patients with nonsense identified, 60 were unrelated. These 60 variants were distributed in 33 of the 79 *DMD* exons, and almost 66% were localized in the dystrophin rod domain. Moreover, in unrelated patients, nonsense mutations were more frequently found in exons 20, 23, 66, and 70. The UGA turned out to be the most frequent premature stop codon observed (47%) and it was in the first position of the codon that 73% of the substitutions occurred, being 2.2 times more frequent transitions than transversions. It is important to highlight that 35% of the 60 unrelated patients carried nonsense mutations involving Arginine and 30% involving Glutamine. Both of them were caused by transitions, and all mutations in the case of arginine, were CGA codons. Of the 20 amino acids encoded in our genetic code, only ten can convert to a stop codon by mutations. We observed nine of them present in our cohort, only glycine was not found (Table 1).

### Meta-Analysis of Latin America’s Metrics for Duchenne Muscular Dystrophy Available Therapies

A total of seven reports (six published manuscripts and a thesis) matched the inclusion/exclusion criteria used for the systematic review of the literature, representing only six Latin American countries (Table 2). Furthermore, in order to compare the information from Latin America with the highly regarded knowledge from Europe and North America, manuscripts from Spain, Italy, Portugal and the United States were included in our meta-analysis (Table 2). Unfortunately, though the manuscript from Portugal included a large cohort and performed the complete diagnostic algorithm, the data submitted in LOVD was incomplete and showed a bias toward small sequence variants.

**TABLE 2:** Meta-analysis of Latin America’s metrics for DMD available therapies.

Country	Total/diagnosed cases^a^	Employed techniques	AD	Exon 45 skipping (E45S)^b^	Exon 51 skipping (E51S)^b^	Exon 53 skipping (E53S)^b^	Ataluren^c^	Reference
Argentina	371/359	MLPA and *DMD* seq. by NGS	6 years	9/155 (5.8%)	20/155 (12.9%)	18/155 (11.6%)	60/143 (42%)	Present work
Brazil	177/177	MLPA and *DMD* seq. by NGS	—	19/103 (18.4%)	11/103 (10.7%)	13/103 (12.6%)	22/52 (42.3%)	de Almeida et al. (2017)
Peru	40/21	PCRm and MLPA	—	4/17 (23.5%)	3/17 (17.6%)	2/17 (11.8%)	—	Huaman-Dianderas et al. (2019)
Colombia	52/52	MLPA and *DMD* seq. by NGS	9 years	1/28 (3.6%)	2/28 (7.1%)	0/28 (0%)	6/11 (54.5%)	García-Acero et al. (2018)
Costa Rica	74/53	PCRm and MLPA	7.5 years	7/45 (15.6%)	8/45 (17.8%)	1/45 (2.2%)	—	Thesis^3^
Puerto Rico	84/65	MLPA	—	1/56 (1.8%)	9/56 (16.1%)	5/56 (8.9%)	—	Ramos et al. (2016)
Mexico	170/116	MLPA, PM-MLPA, HRM and sanger seq	—	11/86 (12,8%)^d^	11/86 (12,8%)^d^	11/86 (12,8%)^d^	—	López-Hernández et al. (2015)
	63/52	MLPA, NGS and sanger seq	—	—	—	—	11/29 (37.9%)	Alcántara-Ortigoza et al. (2019) ^§^
Spain	284/284	PCRm, MLPA and sanger seq	—	11/131 (8.4%)	16/131 (12.2%)	8/131 (6.1%)	49/97 (50.5%)	Vieitez et al. (2017)
Italy	1902/1902	PCRm, Log-PCR, MLPA, NGS and sanger seq	—	39/610 (6.4%)^d^	51/610 (8.4%)^d^	53/610 (8.7%)^d^	200/469 (42.6%)	Neri et al. (2020)
Portugal	312/284	Southern blot, PCRm, MLPA and sanger seq	—	0/11 (0%)^d^	1/11 (9.1%)^d^	0/11 (0%)^d^	5/25 (20%)	Santos et al. (2014)
EUA	933/933	SCAIP, MLPA, sanger seq and cDNA seq	—	53/426 (12.4%)	70/426 (16.4%)	50/426 (11.7%)	226/400 (56.5%)	Flanigan et al. (2009)

a Tslrotal of molecularly analyzed/diagnosed unrelated male patients, members of a family were counted as 1 case; AD: Mean age at diagnosis

b Percentage of deletions amenable with exon skipping of exon 45, 51, and 53, respectively. Calculations were performed as follows: N° of unrelated patients amenable for each of the therapies/N° of unrelated patients carrying deletions

c Percentage of *DMD* small variants candidates for Ataluren or premature stop codon read through. Calculations were performed considering: N° of unrelated patients carrying nonsense variants/N° of unrelated patients with sequence variants. N/A: Data not available.

d Frequencies were determined on the basis of the patients reported on LOVD and linked to the manuscript.

Strikingly, according to the gathered reports, only four Latin American countries (Argentina, Brazil, Colombia and Mexico) conduct the screening of *DMD* small sequence variants, that is to say, the complete molecular diagnostic algorithm for dystrophinopathies (CNV and SNV). Thus, most of the Latin American countries only provide studies to detect CNVs. As it was part of the inclusion criteria, only these seven manuscripts report the usage of MLPA, the rest of the available Latin American literature in PubMed and Google Scholar not only were they more than 15 years old but also entailed studies of multiplex-PCR to detect deletions in the *DMD* gene. On the other hand, information about the age at molecular diagnosis was only provided for three countries, being the earliest the Argentinian at the age of 6 years old and the latest the Colombian at the age of 9 years (Table 2).

Regarding the frequencies of exon skipping, as in the manuscripts they were estimated in alternative ways, we standardized the calculations as: total amount of unrelated patients amenable for each exon skipping/total amount of unrelated patients with *DMD* deletions. We observed different relations among the three targets for exon skipping in the analyzed countries. As it is generally reported in the literature, the pattern E51S > E53S > E45S was detected in Argentina and Puerto Rico, which also coincided with the Spanish results. Alternatively, Colombia and Costa Rica showed the pattern E51S > E45S > E53S, which was shared with the United States. In addition, exon 45 proved to be the most frequent target for exon skipping in Brazil (E45S > E53S > E51S) and Peru (E45S > E51S > E53S), but they presented different relations for exons 51 and 53. Notably, Mexico exhibited the same frequency for the three targets (E45S = E51S = E53S). Similarly, Italy depicted almost the same frequency for exon 51 and 53 skipping, yet a reduced proportion for exon 45 (E51S ≈ E53S > E45S).

Concerning the premature stop codon read-through therapy, we calculated the proportion of nonsense variants as follows: total amount of unrelated patients with nonsense variants/total amount of unrelated individuals with *DMD* small molecular alterations. The proportions of nonsense variants for Latin America were mainly around 40%, which correlated with the results from the Spanish and Italian populations. Colombia exhibited the greater amount of nonsense mutations (54%) among the Latin American countries and was similar to the 56.5% observed in the United States.

## Discussion

Dystrophinopathies cover a spectrum of X-linked muscle diseases ranging from mild to severe phenotypes. Although rare, they are among the most common pediatric muscular dystrophies, being DMD the most prevalent and severe form. Luckily, in the last two decades, unprecedented advances have been made in the field of drug development for rare diseases and DMD is a great example. Nowadays, not only are corticosteroids available, but also mutation-dependent therapies aiming to generate a functional dystrophin mRNA and/or protein. These major advances have turned the molecular diagnosis of dystrophinopathies into a key element for the selection of the best standard of care. In other words, screening and precise characterization of the *DMD* causative mutation is now the basis of the theragnosis for these diseases.

In the present work, we pursued differential molecular diagnosis of 400 Argentinian patients with presumptive clinical diagnosis of dystrophinopathy, so as to determine eligibility for the available therapies. For this, as it was mentioned above, we set up a general diagnostic algorithm following the best practice guidelines for genetic testing for dystrophinopathies (Birnkrant et al., 2018; Fratter et al., 2020), which was tailored on the basis of the particular characteristics of each case. Moreover, this strategy was improved by taking into consideration the possibility of mistaken clinical diagnosis given the existence of overlapping phenotypic features with other types of MDs. Here we have shown that, at least for the Argentinian analyzed cohort, the employed algorithm is highly effective for the detection of the disease causing molecular alterations and for the achievement of differential diagnosis, as we reached a detection rate of 97%.

Dystrophinopathy clinical diagnosis was confirmed by genetic testing in 371/400 (92.8%) patients. Apart from being the foundations of familial genetic assessment, molecular diagnosis plays a key role in the selection of the suitable standard of care for each individual (Birnkrant et al., 2018). Corticosteroids are the recommended standard therapy for DMD, thus molecular confirmation of the diagnosis is essential for starting treatment. Therefore in these 371 DMD confirmed patients, corticosteroid treatment was correctly indicated and validated. The addition of corticosteroids in the standards of care for dystrophinopathy was subject of great debate given their well-known side effects. However, they have extensively proved to ameliorate the inflammation and improve muscle strength, which translates in a delay at the age of loss of ambulation. This is the main reason why their benefits are thought to surpass their side effects. Nowadays, the discussion relies on which corticosteroid is the best for dystrophinopathy patients, Prednisone or Deflazacort (Griggs et al., 2016; Shieh et al., 2018).

On the one hand, the employed molecular algorithm was able to identify 17 clinically misdiagnosed patients. While most of these patients were affected by recessive or dominant forms of limb-girdle muscular dystrophies, one of them had a pathogenic variant in *PHKA1* associated with the X-linked form of muscle glycogenosis. Individuals suffering from the autosomal recessive LGMD2I (*FKRP*) were the most frequently misdiagnosed as DMD. This large proportion of mistaken clinical diagnosis relies on the overlapping signs and symptoms among MDs and the requirement of an experienced neurologist/physician to detect the characteristic features of each clinical picture. Even the highly regarded muscle biopsy is no longer considered as an unequivocal diagnostic test, as it has been shown that patients with absence or decreased levels of dystrophin in the immunohistochemistry can carry molecular alterations in genes encoding for dystrophin-related proteins (Yamamoto et al., 2008). Moreover, it must be highlighted that if we would have followed the recommended molecular algorithm for dystrophinopathies without modifications, the 29 individuals not carrying pathogenic variants in *DMD* would have undergone muscle biopsy. This, therefore, shows that when WES or NGS panels results are available, broadening the screening to genes linked to the development of NMDs is useful not only to reach the differential diagnosis without further tests but also to prevent patients from going through the invasive biopsy procedure.

On the other hand, providing differential diagnosis to the 17 individuals with other forms of MDs was also useful to determine the suitable standard of care and eligibility on gene-transfer therapies. They were prevented from a corticosteroid treatment, as for these diseases there is still no strong evidence for its efficacy (Walter et al., 2013; Albuquerque et al., 2014). Furthermore, six of them could already be determined as candidates for gene-transfer therapies that are under preclinical or clinical tests. Even though these are gene-dependent therapeutic approaches, counting with the precise characterization of the disease causative mutations will allow them to start with these treatments as soon as they are approved and become available, so as to prevent further muscle deterioration.

Given the usage of WES for the screening of small variants, we have identified two patients carrying likely pathogenic/pathogenic variants in *DMD* and in other MDs genes (*CAPN3* and *SYNE1*) associated with both dominant and recessive inheritance patterns. One of them even carried a pathogenic variant and a VUS in *DMD*, being the latter reported as likely pathogenic in LOVD. We are observing this type of cases more frequently as NGS tests are becoming more broadly used for genetic diagnosis. On the one side, these findings highlight the importance of performing a deep genetic analysis so as to provide an accurate theragnosis, given that the selected treatment might not be as effective as it should because of the existence of a second deleterious molecular alteration further affecting the skeletal muscle. On the other side, these observations could explain the phenotypic heterogeneity among dystrophinopathy patients.

Regarding *DMD* mutational spectrum, the observed proportion of CNVs and small sequence variants were in accordance with what was reported in literature for the European and North American populations (Flanigan et al., 2009; Falzarano et al., 2015; Aartsma-Rus et al., 2016; Vieitez et al., 2017). We even detected 0.7% of large allelic del-dups. Notably, ∼7% of the identified gross duplications were non-contiguous alterations.

In particular, 5% of the patients with single exon deletions detected by MLPA, actually had small sequence variants affecting the hybridization of the hemiprobes. Despite the fact of being included in the best practice guidelines for genetic testing for dystrophinopathies, at least in our country, corroboration of single exon deletions with an alternative technique is not the norm for every laboratory. Yet, this has a huge impact for the patient, as a mistaken diagnosis can affect management and theragnosis.

As for the 12 individuals with clinical presumptive diagnosis of dystrophinopathy but without identified pathogenic mutation, we could determine that five of them had a biopsy with immunohistochemistry compatible with dystrophinopathy (dystrophin deficient or absent). Therefore, we presumed they could be dystrophinopathy cases with deep intronic alterations, chromosomal rearrangements or regulatory mutations not detected by the employed methodology (Gurvich et al., 2008; Tran et al., 2013; Aartsma-Rus et al., 2016). Another seven patients had highly increased CK levels (ranging from 1.600 to 18.000 UI/L), however they did not have a biopsy. In conclusion, to continue with the study of these undiagnosed patients complementary studies will be necessary. A muscle biopsy could be valuable to perform Immunohistochemistry analysis, western blot and/or mRNA sequencing. Other options are MRI, electromyography and other genetics tests that analyze genes that were not included in the NMD gene table (new NMD-associated genes) or genes carrying variants not detected by the employed NGS pipeline. Such are the cases of facioscapulohumeral muscular dystrophy (FSHD) or type three spinal muscular atrophy (SMA).

Regarding exon skipping, the selection of candidate patients is generally performed considering the exonic borders of the observed deletion at gDNA level and determining if the removal of one or several of the surrounding exons would restore the reading frame. This way we could establish that 53 of the dystrophinopathy patients apply for the available strategies targeting exons 45, 51, and 53. However, it should be noticed that as the cDNA is not evaluated for these patients, we do not know if the molecular alteration at the mRNA level resembles the one identified in the gDNA. That is to say, it cannot be excluded that, given the location of the intronic breakpoints of the deletion, non-canonical regulatory splicing sites might be altering the mRNA processing. Hence, as this could modify the effect of the exon skipping therapy and make patients go through an ineffective treatment, taking this matter into consideration, would further improve the selection of candidate patients (Gualandi et al., 2006; Tuffery-Giraud et al., 2017).

As for the screening of putative exonic targets for the identified out-of-frame deletions, we observed that the majority of patients (87.5%) would require a mono-target exon skipping strategy to restore their reading frame. Luckily, this agrees with the knowledge obtained from the development of different exons skipping mechanisms, as the mono-target therapies have reached more fruitful results than the multi-target ones. This is related to the great difficulty with the delivery of the required chemically modified AONs, turning the task more laborious as the amount of targets increases (Aslesh et al., 2018; Echigoya et al., 2019). Furthermore, among the most useful exonic targets, we found exons 44, 50 and 52 (∼17.8%) which are already undergoing preclinical trials^2^.

Also, for 14 patients, we identified two different single-exon skipping strategies capable of restoring the reading frame. In such cases the question is which would be better or would be more effective for the patients. We will try to answer this query using as an example the deletion of exons 3 to 7 which is amenable for exon skipping of exon 2 and exon 8. As far as we are concerned, the decision should be made on the basis of the location of the molecular alteration, the role of the implicated area in the protein, information of gene/protein structure and reports of patients having the resulting deletion and their phenotype or clinical course of the disease. In this case, although the deletion affects the actin-binding domain, it is known that there is another actin anchorage site within the rod domain (Mias-Lucquin et al., 2020). Moreover, it has been reported the existence of three Internal Ribosome Entry Sites (IRES) or, in other words, three internal in-frame start codons of the translations in exon 8 (Malhotra et al., 1988; Winnard et al., 1995). In addition, LOVD counted with five reports of patients with deletion of exons 2 to 7 (three classified as BMD, one as DMD and 1 DMD/BMD), while there were only three reports of the deletion of exons 3 to 8 (1 BMD and 2 MD). Finally, according to the provided information, we think that in this case the exon skipping of exon 2 would show better results.

Concerning premature stop codon read-through therapy, given the fact that nonsense variants are generally considered as truncating alterations, candidate patients are selected under the simple consideration of presenting this type of small sequence variants. Following this criteria, we identified in our cohort 70 individuals eligible for the treatment with Ataluren. Nonetheless, this year it was reported that not every *DMD* nonsense variant should be rendered as truncating nor strictly associated with DMD, as due to their genetic/exonic location they could be actually having a milder effect on the phenotype (Neri et al., 2020; Torella et al., 2020).

Granted the existence of a therapeutic protocol for nonsense variants, it is of the utmost importance to deepen the knowledge of the effect of this type of alterations and their characterization. In our cohort, we have found 60 nonsense in unrelated patients, distributed in 33 of the 79 *DMD* exons and mainly affecting the rod domain (65.6%) of dystrophin. These substitutions principally took place in the first codon position (73%), followed by 19% affecting the second and 8% the third position, mostly disrupting codons coding for arginine and glutamine. Also, it was observed that transitions occurred 2.2 times more frequent than transversions, being C > T substitution the most prevalent. Finally, the stop codon generation rate was: UGA (46.9%), UAG (32.8%) and UAA (20.3%).

As expected, G:C > A:T transitions were the most prevalent stop mutation class (72%), we obtained results similar to Flanigan et al., 2009. We also found arginine as the most frequent amino acid converted to stop. From the total of the 60 substitutions, the 35% were transitions due to CpG from arginine (CGA) to Stop, presumably due to the spontaneous deamination of 5-methylcytosine to thymidine at methylated CpG dinucleotides (Cooper and Krawczak, 1989; Flanigan et al., 2009).

Although up to now nonsense mutations are treated with Ataluren, it is possible that in a short term, combined therapies will begin to be implemented. For example, 13 of the 60 nonsense (exons: 9, 10, 15, 16, 23, 27, 30, 32, 34, 39, 48, 60, and 64) are located in inframe exons, these exons could be used as targets for exon skipping and be combined with the premature stop codon read-through therapy.

Another important point to take into account in the trials is that the populations (controls vs. treated) to be compared should be as homogeneous as possible in terms of nonsense variants. Beyond the fact that most nonsense are considered null alleles, it must be taken into account that their location in the *DMD* gene will have different functional impact since it will depend on how many isoforms will be affected. In our case, patients who have nonsense after exon 60 will theoretically have all the isoforms of dystrophin affected. On the other hand, as more 5′ the nonsense would be located, less isoforms would be altered. Regarding the above mentioned nonsense characteristics, although 10 patients are currently being treated with Ataluren, unfortunately, we do not have enough clinical data to make genotype-phenotype statistical comparisons to determine the effectiveness of the treatment.

In the matter of the performed meta-analysis, it surprised us the lack of updated reports about the genetic and molecular characterization of dystrophinopathy patients from Latin America. Moreover, it is alarming the reduced number of countries providing the *DMD* full mutational analysis to the affected individuals, which, as it was highlighted throughout the manuscript, is now considered the foundations of the theragnosis for dystrophinopathies. However, we cannot discard the chance that these studies are in fact carried out but the results are not shared in publications. Another possibility is that the studies are performed abroad, as it is well-known that for developing countries sometimes it is cost-effective to send the samples to experienced and equipped laboratories.

In addition, it was difficult to obtain information regarding the age at molecular diagnosis, though manuscripts more frequently detailed the age at onset. Nonetheless, we reckon that the age at molecular diagnosis is more objective and precise than the age at onset, as the latter depends on the ability of the family to detect the appearance of the first symptoms and the capacity of the clinician to get the information during anamnesis. On the other hand, the age at molecular diagnosis provides insights about the availability of genetic studies in a certain place. It must be noticed that an early confirmation of the presumptive clinical diagnosis has a major impact on the management and treatment of the patient, as this translates into a major amount of healthy muscle fibers to treat. From the gathered data, Argentina presented the earliest age at molecular diagnosis (6 years) while Colombia showed the latest one (9 years). So, efforts must be done in Latin America to achieve early diagnosis in dystrophinopathy patients.

As for exon skipping, not only has the meta-analysis depicted an ample variability in the frequencies for each target exon, but also has demonstrated the existence of different patterns among them. This was true for the Latin American and European countries, proving that there is no such thing as a general continental pattern. Furthermore, as it has been reported for the Italian population, there can even exist differences in the exon skipping frequencies within a country (Neri et al., 2020). Hence, these results suggest that the selection of target exons for the development of exon skipping therapies, based on frequencies rendered as “general” in the literature, might not be the best approach. As local/ethnic differences are not being considered, many dystrophinopathy patients carrying frequent molecular alterations might miss the opportunity to access a mutation-dependent therapy suitable for them.

On the other hand, the obtained proportions of nonsense variants were similar in the analyzed countries, not even displaying differences between continents. The majority of the countries presented frequencies around 40%. However, Colombia and the United States showed an increased proportion of nonsense mutations, reaching ∼55%. Thus, this information suggests an even distribution of this type of small variants among different populations.

In conclusion, the present manuscript describes the theragnosis carried out in one of the reference centers for the molecular diagnosis of dystrophinopathies in Argentina. Firstly, the implemented diagnostic molecular algorithm proved to be efficient for the achievement of differential diagnosis, which nowadays plays a crucial role in patient management, the determination of the suitable standard of care and genetic counseling. Secondly, we have performed a thorough characterization of the *DMD* molecular alterations and, particularly, of the nonsense variants observed in an Argentinian dystrophinopathy cohort. Thirdly, we conducted a meta-analysis that allowed us to compare the frequencies of the amenable mutations for the available DMD therapies and the current situation of the dystrophinopathy molecular studies throughout Latin America. Finally, this work contributes with the international efforts to characterize the frequencies and variants of Latin America, pillars of drug development and theragnosis.

## Data Availability

The datasets presented in this study can be found in the LOVD database, accession link: https://databases.lovd.nl/shared/variants/in_gene#object_id=Transcript%2CVariantOnTranscript%2CVariantOnGenome&id=0&order=geneid%2CASC&search_VariantOnGenome/Reference=luce%202021&page_size=100&page=1.

## Footnotes

1
https://www.sarepta.com/science/gene-therapy-engine.

2
https://www.sareptatherapeutics.ch/en/our-pipeline.
